# Foliar Selenium Application Enhances Wheat Resistance to *Bipolaris sorokiniana*-Induced Spot Blotch via Modulation of Growth, Physiological Homeostasis, and Antioxidant Defense Systems

**DOI:** 10.3390/life16081353

**Published:** 2026-08-17

**Authors:** Muhammad Raheel, Hafiz Muhammad Usman Aslam, Saba Aslam, Waqas Ashraf, Kamran Ikram, Tahira Abbas, Muhammad Zeeshan Mansoor, Qamar uz Zaman, Muhammad Umar Alvi, Sajjad Ahmad, Hafiz Muhammad Aatif

**Affiliations:** 1 Department of Plant Pathology, Faculty of Agriculture and Environment, The Islamia University of Bahawalpur, Bahawalpur 63100, Pakistan; 2 Plant Pathology Program, San Luis Valley Research Center, Colorado State University, 249 E County Road 9 N, Center, CO 81125, USA; 3 Department of Plant Pathology, Muhammad Nawaz Shareef University of Agriculture, Multan 60000, Pakistan; 4 Department of Plant Pathology, University of Agriculture, Faisalabad 38000, Pakistan; 5 Department of Agricultural Engineering, Khwaja Fareed University of Engineering and Information Technology, Rahim Yar Khan 64200, Pakistan; 6 Department of Horticulture and Plant Pathology, Faculty of Agricultural Sciences & Technology, University of Layyah, Layyah 31200, Pakistan; 7 Department of Plant Pathology, Bahauddin Zakariya University, Multan 60800, Pakistan; 8 Department of Environmental Sciences, The University of Lahore, Lahore 54000, Pakistan; 9 Department of Soil Science, Faculty of Agricultural Sciences & Technology, University of Layyah, Layyah 31200, Pakistan

**Keywords:** *Bipolaris sorokiniana*, foliar application, growth, physio-chemical, selenium biofortification, stress

## Abstract

Spot blotch has become a major devastating disease in wheat. In the current study, Faisalabad-08 was supplemented with various levels of selenium (Se) both in vitro and in greenhouse experiments (CRD, *n* = 3) to counter the spot blotch pathogen. The results revealed that minimum disease incidence was assessed in the case of T_2_ (50 mg L^−1^). However, maximum plant growth attributes, including plant height (PH), plant fresh weight (PFW), plant dry weight (PDW), leaf surface area (LSA), and root length (RL), were recorded in T_5_ (50 mg L^−1^ + pathogen). Similarly, chlorophyll a, chlorophyll b, total chlorophyll, membrane stability index, carotenoid, relative water contents, proline, sugar, flavonoid, total phenolic content, SOD, POD, CAT, PPO, and PAL contents were enhanced in T_5_ compared to other tested treatments. However, MDA, an oxidative damage marker, was significantly decreased with T_5_. Correlation, PCA, and heatmap analysis suggested that all the attributes were significantly interrelated, except MDA, in defining the crop’s potential to sustain its growth under biotic stress. In crux, foliar Se application (50 mg L^−1^) effectively mitigates spot blotch through enhanced antioxidant defense and physiological homeostasis. This sustainable approach offers a viable alternative to conventional fungicides for integrated wheat disease management.

## 1. Introduction

In the current era of industrialization and globalization, a sustainable food supply for an exponentially growing human population has become a major concern [1]. Wheat is among the most important cereal food crops, and is a key part of the human diet around the globe, including Pakistan [2]. The crop contributes around 2.2% to the gross domestic product and 10% in value-added products of Pakistan. The food security of a country is highly dependent on a sufficient wheat supply throughout the year [3]. However, the sustainability and yield potential of different wheat-growing agro-ecosystems have been largely compromised due to the proliferation of several phytopathogenic fungi, including *Helminthosporium*, *Botrytis*, *Rhizoctonia*, *Fusarium*, *Alternaria*, and *Bipolaris* species [4,5,6,7,8,9].

Among different plant pathogenic diseases, spot blotch has emerged as a serious threat to wheat in the last four decades [10]. The effect of the disease becomes more severe when the post-anthesis phase coincides with an environment of high relative humidity and high temperature for a long period. The disease infects nearly 25 M ha (16–43%) of wheat crops all around the globe; 40% of this area lies within the territories of the Indo-Pak subcontinent [11]. The spot blotch pathogen causes 15–20% yield losses in this area [12]. Disease not only causes reductions in terms of yield but also deteriorates the quality of harvested grains for end users [13]. 

Previously, it was determined that spot blotch disease occurs due to a complex of three different fungal species: *Alternaria triticina*, *Pyrenophora tritici repentis,* and *Helminthophobia sativum* [14]. However, recently, it has been reported that the *Bipolaris sorokiniana* pathogen is the actual pathogen of spot blotch disease. This pathogen also causes head blight, seedling blight, root rot, and black points in wheat [15]. The infection starts with light brown oval- to elliptical-shaped colored lesions that first appear on the leaves, nodes, glumes, and sheaths of crop plants. As the pathogen infection progresses, the leaf lesions gradually increase in size and the whole leaf becomes chlorotic, later turning brown and dry. The infection also interferes with the leaf’s photosynthetic capacity and decreases the grain-filling stage of the wheat crop [14]. The pathogen usually transmits through infected seeds, crop stubble, and soil, and secondary infection may take place through air [15]. However, the disease severity is influenced by several factors, including the soil fertility level, crop management techniques, crop developmental stages, and prevailing climatic conditions [16]. High relative humidity favors disease incidence, especially in areas with day temperatures greater than 18 °C [17]. Moreover, the pathogen infection spreads exponentially in the Indian subcontinent at 26 °C, which explains why late-sown wheat is susceptible to disease attack [18].

The rising concern regarding the management of plant diseases with traditional chemicals raises serious concerns about environmental quality, food safety, and resistance development, which necessitates the immediate exploration of certain eco-friendly alternative approaches for managing plant phytopathogens [19]. Enhancing plant resistance against different biotic and abiotic stressors with adequate mineral nutrition is a cost-effective and bio-compatible strategy. Mineral nutrition has vital roles in plant disease resistance or tolerance and also plays important functions in regulating plant growth and development, particularly under stressful conditions [20].

Selenium (Se) is an essential mineral for humans, animals, and plants at low concentrations [21,22]. Its application as a base fertilizer or foliar spray on plants is reported in several studies [23]. In soil as sodium selenate (Na_2_SeO_4_), it is also in practice at Finnish farms [24]. It can exist in many organic and inorganic oxidation forms, which are exchangeable with each other biochemically or chemically [25]. It is also present in soil as inorganic compounds such as selenite (SeO_3_^2−^) and selenate (SeO_4_^2−^). Plants absorb Se in the form of selenite, which is present abundantly in soil [26]. After being converted to selenite and selenide, this selenate is subsequently added to amino acids as selenocysteine and selenomethionine (SeMet) [27,28]. At a low dosage, it is good for plant growth, while, a at high dosage, it acts as a pro-oxidant and exhibits plant damage [29,30]. It is volatilized as dimethyl selenide in plants [31]. It is reported for its accumulation and transformation in bioactive molecules having nutraceutical tendencies [32], which declare it an essential element for plant growth [25]. It also improves plant resistance against abiotic stresses (such as plant stress due to the accumulation of heavy metals) and drought tolerance. Further, it improves the plant capacity against ultraviolet B, low and high temperature, salt senescence, and desiccation [30]. It was observed that the appropriate application of Se at an appropriate concentration improves the fungitoxic activity against different fungal pathogens and bacteria [21]. Therefore, Se is a good alternative for plant-disease control, particularly against fungal diseases.

While selenium’s role in abiotic stress tolerance is well-documented [29,30], its specific mechanisms against *B. sorokiniana* remains poorly characterized. Furthermore, most studies focus on either in vitro antifungal activity or greenhouse responses in isolation. This study integrates both approaches, providing comprehensive evidence for Se-mediated defense priming through enzymatic and non-enzymatic antioxidant pathways. We hypothesized that foliar Se application would (1) directly inhibit *B. sorokiniana* growth in vitro, and (2) enhance wheat resistance through the coordinated upregulation of physiological and biochemical defense mechanisms, with 50 mg L^−1^ being the optimal concentration.

## 2. Materials and Methods

### 2.1. Study Area

The trials were carried out in the laboratory and greenhouse facilities at The Islamia University of Bahawalpur, Punjab, Pakistan (29.3979° N, 71.6726° E; 117 m altitude). The greenhouse maintained 25 ± 2 °C temperature, and 65–70% relative humidity, having a light/dark photoperiod of 14/10 h (400 μmol m^−2^ s^−1^ of photosynthetically active radiation).

### 2.2. Antifungal Assay

#### 2.2.1. Sample Collection, and Isolation and Identification of Fungi 

Leaves of wheat exhibiting distinct spot blotch symptoms, viz., small brown to large scattered necrotic areas, were collected from the wheat fields located at the research field area of The Islamia University of Bahawalpur, Punjab, Pakistan. The collected leaves were subjected to surface sterilization using a NaOCl solution (2%) and ethanol (70%). After this, these leaves were rinsed using sterile distilled water. Using sterile filter paper, these leaves were then dried, sliced into small pieces, and put on petri dishes. The Petri plates contained Potato Dextrose Agar (PDA) medium. This PDA medium was synthesized by mixing 39 g PDA in 1000 mL distilled water and then autoclaved for 15 min at 121 °C. Clindamycin, an antibacterial drug, was mixed to the PDA medium at 0.2 mL per 100 mL. Parafilm tape was used to cover the Petri plates in a way to avoid impurities. The plates were incubated at 28 °C for a period of 5 days to allow the fungus to propagate under controlled conditions. To acquire pure cultures of the pathogen, colonies of the fungus that developed were transferred on fresh PDA plates [33]. To purify the fungus, a single spore of *Bipolaris sorokiniana* fungi was then carefully placed onto a potato dextrose agar medium [34]. Subsequently, slides were prepared for fungal identification.

Single spore isolation was used to obtain a pure culture. The fungus was identified as *Bipolaris sorokiniana* by its characteristic colony and microscopic features: olive to dark-brown colonies, geniculate conidiophores with brown, oblong to cylindrical, multicelled conidia with rounded ends.

#### 2.2.2. Evaluation of Antifungal Activity of Se

Sodium selenite (Na_2_SeO_3_, Sigma-Aldrich, St. Louis, MO, USA, ≥98% purity) was prepared in sterile distilled water at concentrations of 100, 150, 200, 250, and 300 μg mL^−1^. Barresten^®^ (Barrett Hodgson, Karachi, Pakistan) served as the positive control. A 6 mm-diameter mycelial plug from a 72 h-old *B. sorokiniana* culture was placed centrally on PDA plates amended with respective Se concentrations. A digital Vernier caliper (Mitutoyo, Kawasak, Japan) was used to measure the mycelial growth diameter after the plates were cultured for seven days at 25 ± 1 °C.

#### 2.2.3. Greenhouse Experiments

A greenhouse-based study was carried out from November 2024 to April 2025 to examine the possible antifungal effects of sodium selenite (Na_2_SeO_3_) against *Bipolaris sorokiniana*. Sterilized soil (sandy loam soil with a composition of 40% clay, 20% silt, and 40% sand) was put into soil pots with a 10 kg capacity. The wheat variety selected for the experiment was Faisalabad-08, known for its susceptibility to this disease, obtained from the Wheat Research Institute, Ayub Agricultural Research Institute (AARI), Faisalabad, Pakistan. The seeds were subjected to surface sterilization using a 0.1% mercuric chloride solution following the protocols as described by Abdel-Moneim et al. [35]. To ensure a homogenous experimental setup, a completely randomized design (CRD) was adopted with three replicates. The experiment started with the application of low concentrations of Na_2_SeO_3_ via foliar spray, which were progressively increased to assess the effect of the Na_2_SeO_3_ on suppressing the *B. sorokiniana* growth. Each sodium selenite concentration was applied fifteen days after pathogen application in two splits with a seven-day gap.

#### 2.2.4. Inoculum Preparation and Application

*Bipolaris sorokiniana* fungi was isolated from wheat plant leaves and subsequently cultured on PDA media. To get suitable fungal growth, plates with inoculated agar for ten days were incubated at 23 °C. To prepare pure conidial cultures, 12-day-old fungal cultures were scrubbed and mixed with autoclaved distilled water. The resulting fungal inoculum was then placed at room temperature in a shaker, yielding a concentration of 8 × 10^3^ conidia mL^−1^ using a hemocytometer (Neubauer, Lauda-Königshofen, Germany). The resulting conidia were dispersed in Tween-20 solution [36]. 

At the booting stage (Zadoks growth stage 45–49), 50 mL of conidial suspension (8 × 10^3^ conidia mL^−1^) was sprayed uniformly onto each plant using a hand-held atomizer until run-off. Plants were covered with clear polyethylene bags for 48 h and misted with distilled water every 6 h to maintain high humidity for infection. Environmental conditions in the greenhouse were maintained at 25 ± 2 °C with 80–90% relative humidity. Data was recorded after five-days of fungus application and, later, after every five-day interval (10, 15, 20, 25, and day 30) to monitor the development and progression of the fungal infection on the wheat plants. The overall experimental layout is given in Table 1.

#### 2.2.5. Disease Severity Evaluation

For spot blotch disease severity assessment, wheat leaves were randomly selected from various pots, and this process was conducted in triplicate to ensure reliable results. The severity of the disease symptoms was visually evaluated (Table 2).

To quantify the malady severity on wheat crop under greenhouse conditions, a standard scale (0 to 5) as outlined by [35] was used after ten days of pathogen inoculation. Using the standard Equation (1) by [37], disease severity index (%) was calculated. The scale was used a systematic grading of the disease symptoms, with 0 indicating no symptoms and 5 representing the severe infestation of the disease.

Disease severity index was calculated using Equation (1) [37]:

(1) $$Disease\;severity\;Index\;(\%)=\frac{\sum (Disease\;rating\times Number\;of\;leaves\;in\;each\;rating)}{Total\;no.\;of\;leaves\;\times Maximum\;disease\;rating}\times 100$$

#### 2.2.6. Sample Collection

Random samples were collected, after twenty days of Se application, to obtain representative samples from each treatment in triplicates. These samples were utilized to assess various plant growth parameters (RL, PL, PFW, and PDW), physiological parameters (carotenoid contents, relative water contents, chlorophyll contents, and membrane stability index), and biochemical parameters (sugar contents, proline contents, phenolic contents and flavonoid contents, SOD, POD, CAT, PPO, PAL, and MDA) under disease stressed conditions.

### 2.3. Growth and Biomass Attributes

The height of plant was assessed by using a standard scale to determine the vertical length of the plants. To evaluate the leaf surface area at the flag-leaf stage, a leaf area meter was used. Shoot and root fresh weights were recorded for all treatments, and, subsequently, the same plants were dried for seven days at 65 °C in an oven. The purpose of this drying was to record the dry weight of the plant material using the approach described by Iqbal et al. [38].

### 2.4. Physiological Attributes

The plant chlorophyll content was determined using spectrophotometer (UV-1201, Shimadzu Corporation, Kyoto, Japan). Following the standard procedure reported by Bruinsma [39], two grams of grounded plant leaves were mixed with (80%) acetone (10 mL) and the resulting solution was filtered. The absorbance measurements were taken at three different wavelength levels (645 nm, 652 nm, and 663 nm). Similar methodology was employed to measure carotenoid contents with absorbance wavelength of 480 nm. The calculation of the chlorophyll content was performed using the following equations, Equations (2)–(4):

(2) $${Ch}_{a}=\left(A_{663}\;\times 12.7\right)-\left(A_{645}\times 2.7\right)$$

(3) $${Ch}_{b}=\left(A_{645}\times 22.9\right)-\left(A_{663}\times 4.7\right)$$

(4) $${Ch}_{Total}=\left(\frac{1000\;\times \;A_{652}}{34.5}\right)$$

Relative water content (RWC) in wheat leaves was determined following the standard methodology reported by Mullan and Pietragalla [40]. For leaf fresh weight, leaves from wheat plants were submerged in distilled water for a full day and then removed, and weight of leaves were recorded as turgid weight (TW). Then, leaves were kept in an oven with 70 °C for one week to remove moisture from the leaves. The dried leaves were then weighed again. RWC in leaves was calculated using Equation (5).

(5) $$RWC\;\left(\%\right)=\;\frac{Fresh\;Weight-Dry\;Weight}{T\;W-Dry\;\;Weight\;}\times 100$$

A representative leaf weighing 100 mg was carefully chosen for each sample and cut into tiny discs. The leaf discs were washed with distilled water. The leaf discs were placed into separate test tubes which were positioned in a water bath at 40 °C temperature. An EC meter was used to assess the test tubes’ initial electrical conductivity (C1) following a 30 min incubation period. Following the initial measurement, the test tubes were subjected to an elevated temperature of 100 °C within the water bath for a duration of ten minutes to calculate electrical conductivity (C2) again, as per reported protocols of Sairam et al. [41]. Equation (6) was utilized to determine MSI:

(6) $$MSI=1-\frac{C1}{C2}$$

### 2.5. Plant Biochemical Parameters

A 3% sulfosalicylic acid extraction solution was used to crush 0.2 g of wheat leaves. The ninhydrin reagent and two milliliters of glacial acetic acid were then added to different test tubes along with two milliliters of the leaf extract. In a water bath, the mixture was heated and stirred well to allow the reaction to occur until the color change was noted, and then allowed to cool. The mixtures were then cooled. Four milliliters of toluene were added to each, and thoroughly shaken, after which a distinct colored layer was visible. The colored layer was then transferred to another test tube where its absorbance at 520 nm [42] was recorded. Total proline contents were computed by using Equation (7):

(7) $$TPC\;\left(\frac{\mu \;g}{mL}\right)=\;\frac{Sample\;Absorbance\;\times Dilution\;Factor\;\times K\;value}{Plant\;Tissue\;Fresh\;Weight}$$

The standard procedure reported by [43] was used to measure the soluble sugar contents (SSC) at 490 nm absorbance. Soluble sugar contents were computed by using Equation (8):

(8) $$Soluble\;Sugar\;Contents=\;\frac{leaf\;Sample\;Absorbance\;\times Dilution\;Factor\;\times K\;value}{Fresh\;weight\;of\;Plant}$$

The standard procedure outlined by [44] was used to calculate the total phenolic content. Ultimately, the sample solution’s absorbance at 725 nm was measured using a spectrophotometer (UV-1201, Japan).

The total flavonoid contents were measured by following the protocol given by Hussein et al. [44] and the absorbance of the solution was then measured at 415 nm in a spectrophotometer (UV-1201, Japan).

The standard method suggested by Giannopolitis and Ries [45] was used to quantify the activity of the enzyme superoxide dismutase (SOD). The absorbance of the test reaction mixes at 560 nm was deducted from the absorbance of the non-irradiated reaction mixture, which was used as a control.

To find out the CAT activity reaction solution was prepared using 50 mM phosphate buffer with pH of 7.0, 10 μL of total protein extract, and 3% H_2_O_2_. The CAT activity was calculated by finding the disappearing rate of H_2_O_2_ for 3 min [46].

The activity of phenylalanine ammonia-lyase (PAL) was measured according to the standard procedure as mentioned by Berner et al. [47]. Total protein extract was added to the reaction mixture, which also included 150 mM L-phenylalanine, to give a final volume of 3 mL in borate buffer. This mixture was incubated at 40 °C for 30 min. The PAL activity was then determined by following the production of trans-cinnamic acid at 290 nm with a spectrophotometer.

The polyphenol oxidase (PPO) activity was measured using the usual methods described by Raymond et al. [48]. The reaction mixture comprised of 2.50 mL of 200 mM potassium phosphate buffer (pH 7.0), 200 µL of 20 mM pyrogallol, and 20 µL of extract of all protein. Using a spectrophotometer, the absorbance at 430 nm was measured every five minutes to track the development of the reaction.

By measuring the oxidation of guaiacol in the presence of H_2_O_2_ at 470 nm with an extinction value of 26.6 mM^−1^ cm^−1^, the peroxidase (POD) activity was determined. In a total volume of 3 mL, the standard reaction mixture contained 50 mM potassium phosphate buffer (pH 7.0), 20.1 mM guaiacol, 12.3 mM hydrogen peroxide (H_2_O_2_), and enzyme extract [49].

The amount of malondialdehyde (MDA) was measured by thiobarbituric acid (TBA) method [50]. The absorbance of the supernatant was then determined spectrophotometrically at 532 nm and nonspecific turbidity was subtracted to obtain absorbance at 600 nm. The extinction coefficient of 155 mM^−1^ cm^−1^ was taken for the calculation of MDA concentration.

### 2.6. Statistical Analysis

Each pot was an experimental unit and all experiments were performed in a completely randomized design with three replicates of biological material. The data were analyzed by Fisher’s analysis of variance (ANOVA) with the use of Statistix software (version 8.1, Analytical Software, Tallahassee, FL, USA). After a significant ANOVA, Least Significant Difference (LSD) test was conducted to assess the differences among the treatment means at *p* ≤ 0.05 significance level [51]. The factoextra, corrplot, and pheatmap packages of R software (version 4.2.1; R Core Team, Vienna, Austria) were used for the principal component analysis (PCA) and Pearson correlation analysis and the hierarchical clustering heatmap analysis. Three biological replicates have been performed for all results and are given as mean ± SE.

## 3. Results

### 3.1. Antifungal Assay

**Figure 1:**
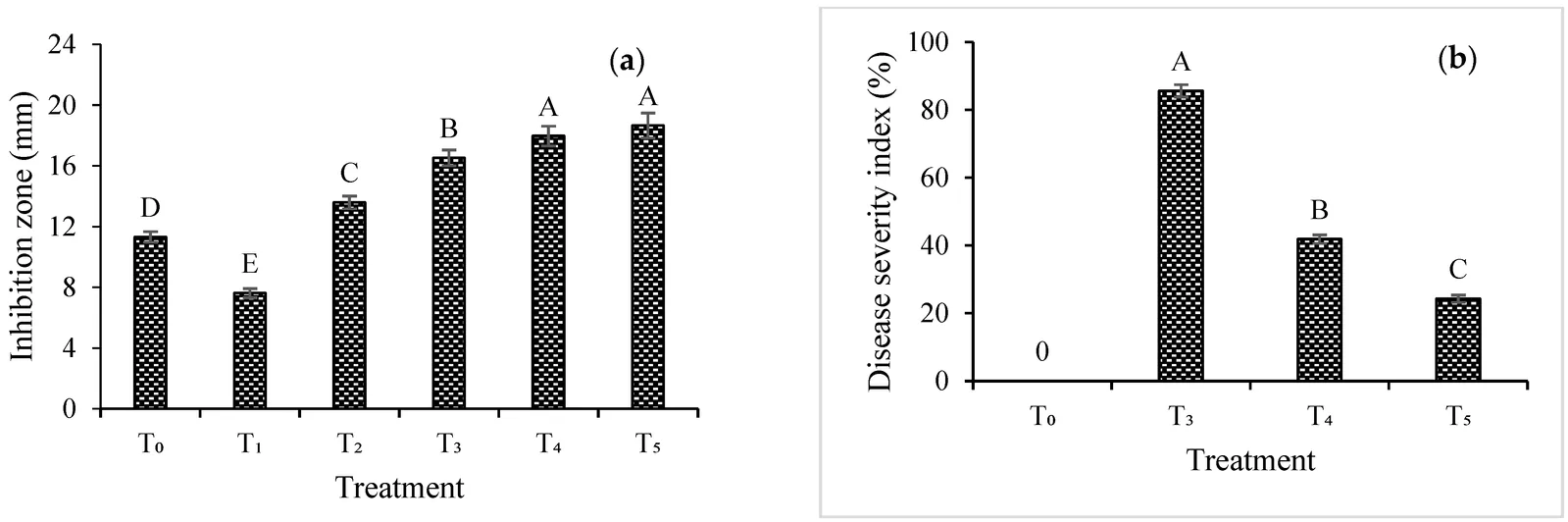
(**a**) Impact of various concentrations of selenium on inhibition zone of *B. sorokiniana* in wheat. Here, T_0_ = Barresten, T_1_ = 100 μg mL^−1^, T_2_ = 150 μg mL^−1^, T_3_ = 200 μg mL^−1^, T_4_ = 250 μg mL^−1^, and T_5_ = 300 μg mL^−1^. (**b**) Impact of various concentrations of selenium on disease severity of *B. sorokiniana* in wheat. Here, T_0_ = No fungus application, T_3_ = No Se application, T_4_ = Fungus + 25 mg L^−1^ selenium, and T_5_ = Fungus + 50 mg L^−1^ selenium.

The results regarding the antifungal activity of Se were compared with an antifungal drug (Barresten). The inhibition zone diameter of the fungus was more pronounced at higher Se concentrations. A higher inhibition zone diameter was obtained in the case of T_5_ (18.65 mm), followed by T_4_ (17.96 mm), T_3_ (16.52 mm), T_2_ (13.59 mm), and T_1_ (7.62 mm), whereas T_0_ (Barresten) exhibited a 11.31 mm inhibition zone diameter (Figure 1a).

### 3.2. Assessment of Disease Severity Index (%)

Disease symptoms were assessed on a visual basis. The results further showed that T_3_ showed a higher disease severity index (85.62%), whereas the minimum severity score was recorded in the combined application of pathogen + selenium (25, 50 mg L^−1^), i.e., T_4_ (41.85%) and T_5_ (24.28%). The disease severity index was not observed in T_0_ (without pathogen application) (Figure 1b).

### 3.3. Growth and Biomass Attributes

**Figure 2:**
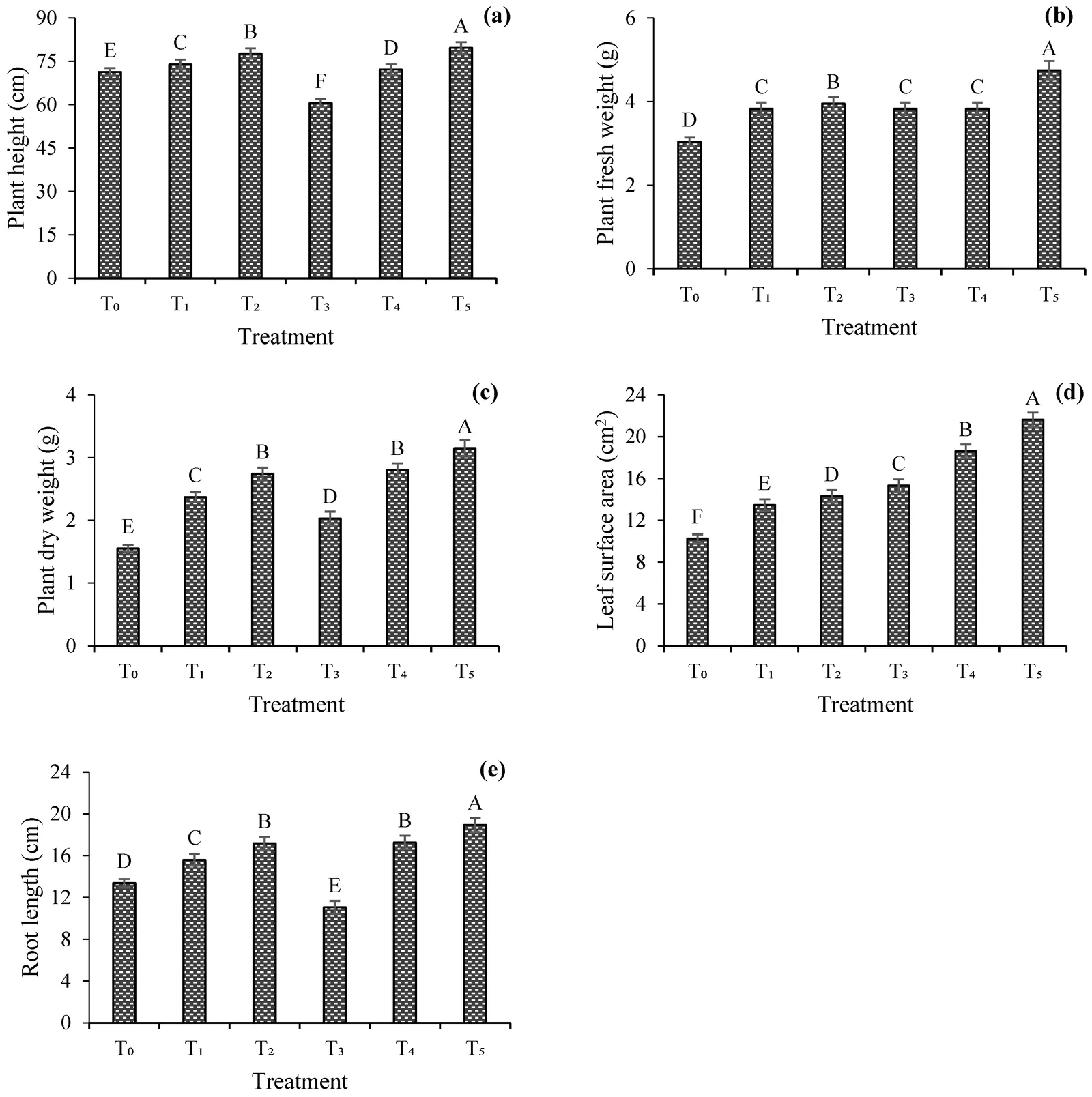
Impact of various treatments on the growth attributes, viz., (**a**) plant height, (**b**) plant fresh weight, (**c**) plant dry weight, (**d**) leaf surface area, and (**e**) root length of wheat under stress regime. T_0_ = Control, T_1_ = 25 mg L^−1^ selenium, T_2_ = 50 mg L^−1^ selenium, T_3_ = Fungal inoculated wheat plants, T_4_ = Fungus + 25 mg L^−1^ selenium, and T_5_ = Fungus + 50 mg L^−1^ selenium.

The growth and biomass attributes of wheat plants were significantly (*p* ≤ 0.05) impacted by the individual (T_1_–T_3_) as well as combined applications (T_4_–T_5_) of selenium and *B. sorokiniana* over control (T_0_). Regarding the sole and combined application of fungus + selenium concentrations (25 and 50 mg L^−1^), T_5_ exhibited the maximum plant height (79.71 cm), representing a 31.6% increase over T_3_ (60.57 cm). The maximum plant fresh weight (g) was observed in the case of T_5_ (4.75 g) over T_0_ (3.04 g) as compared to all tested treatments. In the case of the plant dry weight (g), T_5_ showed the maximum plant dry weight (3.15 g) and T_0_ exhibited the minimum plant dry weight (1.55 g) with respect to other tested treatments. The leaf surface area was higher in the T_5_ (21.63 cm^2^) application, whereas T_0_ showed a lower response (10.26 cm^2^) in this regard over other tested treatments. Regarding the root length (cm), the maximum root length was observed in case of T_5_ (18.94 cm) and a lower value of the root length was assessed in T_3_ (11.06 cm) (Figure 2).

### 3.4. Physiological Attributes

**Figure 3:**
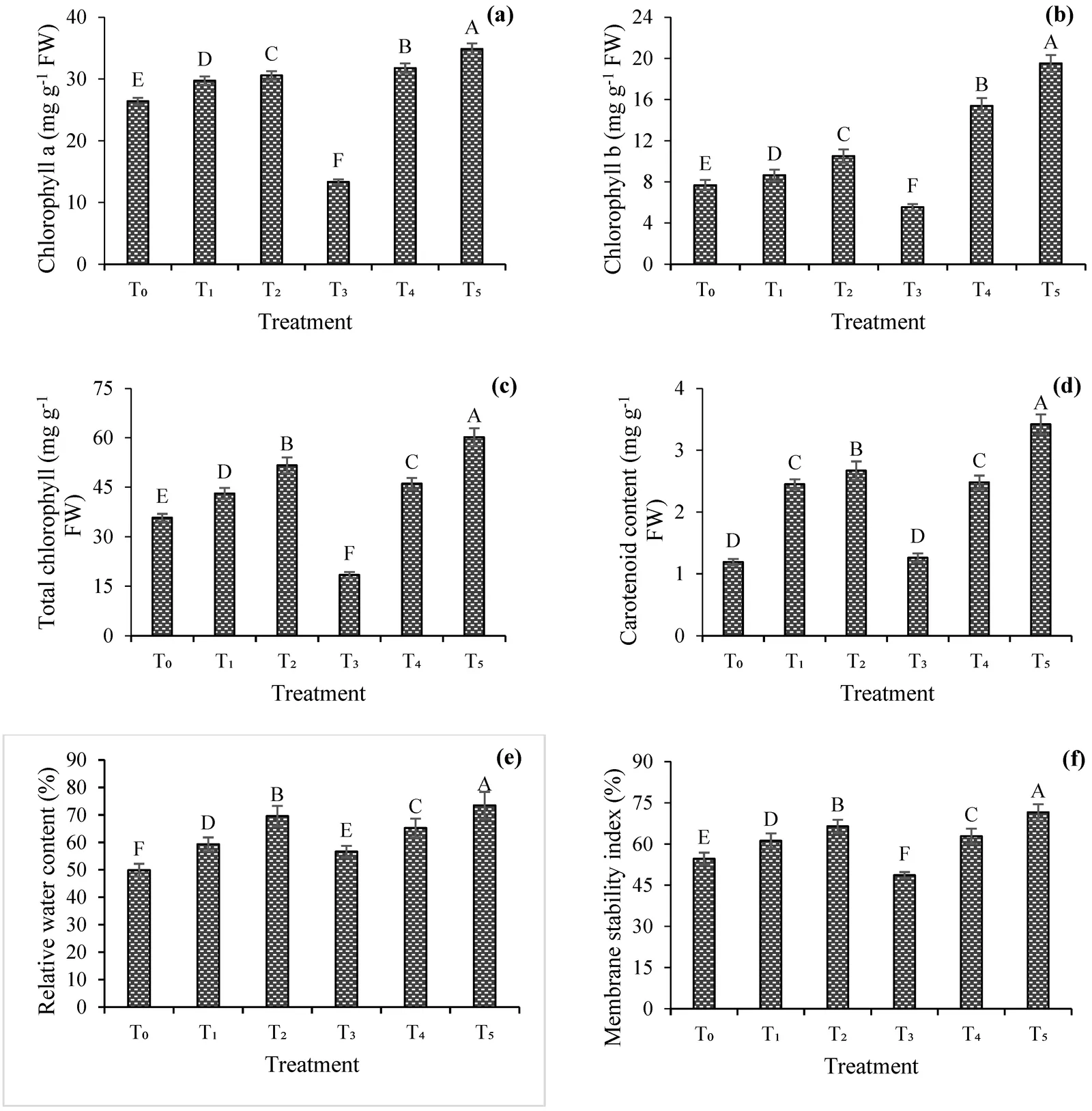
Impact of various treatments on the physiological attributes, viz., (**a**) chlorophyll a, (**b**) chlorophyll b, (**c**) total chlorophyll, (**d**) carotenoid contents, (**e**) relative water contents, and (**f**) membrane stability index of wheat under stress regime. T_0_ = Control, T_1_ = 25 mg L^−1^ selenium, T_2_ = 50 mg L^−1^ selenium, T_3_ = Fungal inoculated wheat plants, T_4_ = Fungus + 25 mg L^−1^ selenium, and T_5_ = Fungus + 50 mg L^−1^ selenium.

The sole (T_1_–T_3_) as well as the combined application of fungus + selenium concentrations (T_4_–T_5_) significantly (*p* ≤ 0.05) impacted the physiological attributes of wheat plants. Regarding the chlorophyll contents (mg g^−1^ FW), T_5_ exhibited the maximum values of chlorophyll a (34.87 mg g^−1^ of FW), chlorophyll b (19.51 mg g^−1^ of FW), and total chlorophyll (60.24 mg g^−1^ of FW), whereas the minimum contents of chlorophyll a (13.34 mg g^−1^ of FW), chlorophyll b (5.54 mg g^−1^ of FW), and total chlorophyll (18.41 mg g^−1^ of FW) were observed in T_3_ (sole fungal inoculated wheat plants). The maximum carotenoid contents (mg g^−1^ of FW) were observed in T_5_ (3.42 mg g^−1^ of FW), whereas their lowest values were recorded in T_0_ (1.19 mg g^−1^ of FW). Regarding the relative water contents (%), T_5_ showed higher relative water contents (73.45%) over other tested treatments. The membrane stability index (%) was higher in the case of T_5_ (71.54%), whereas the minimum membrane stability index was observed in T_3_ (48.64%) (Figure 3).

### 3.5. Biochemical Attributes

**Figure 4:**
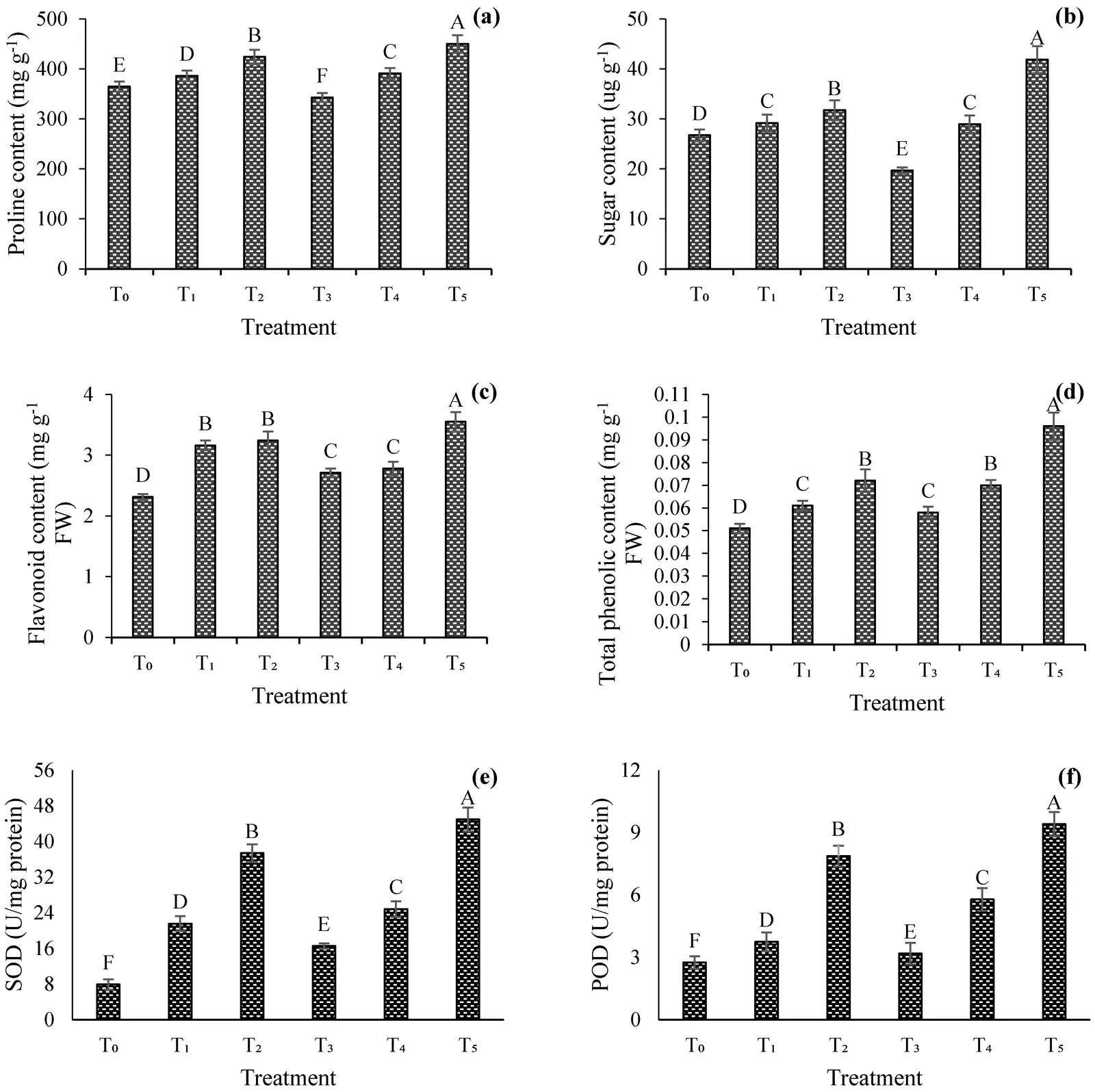

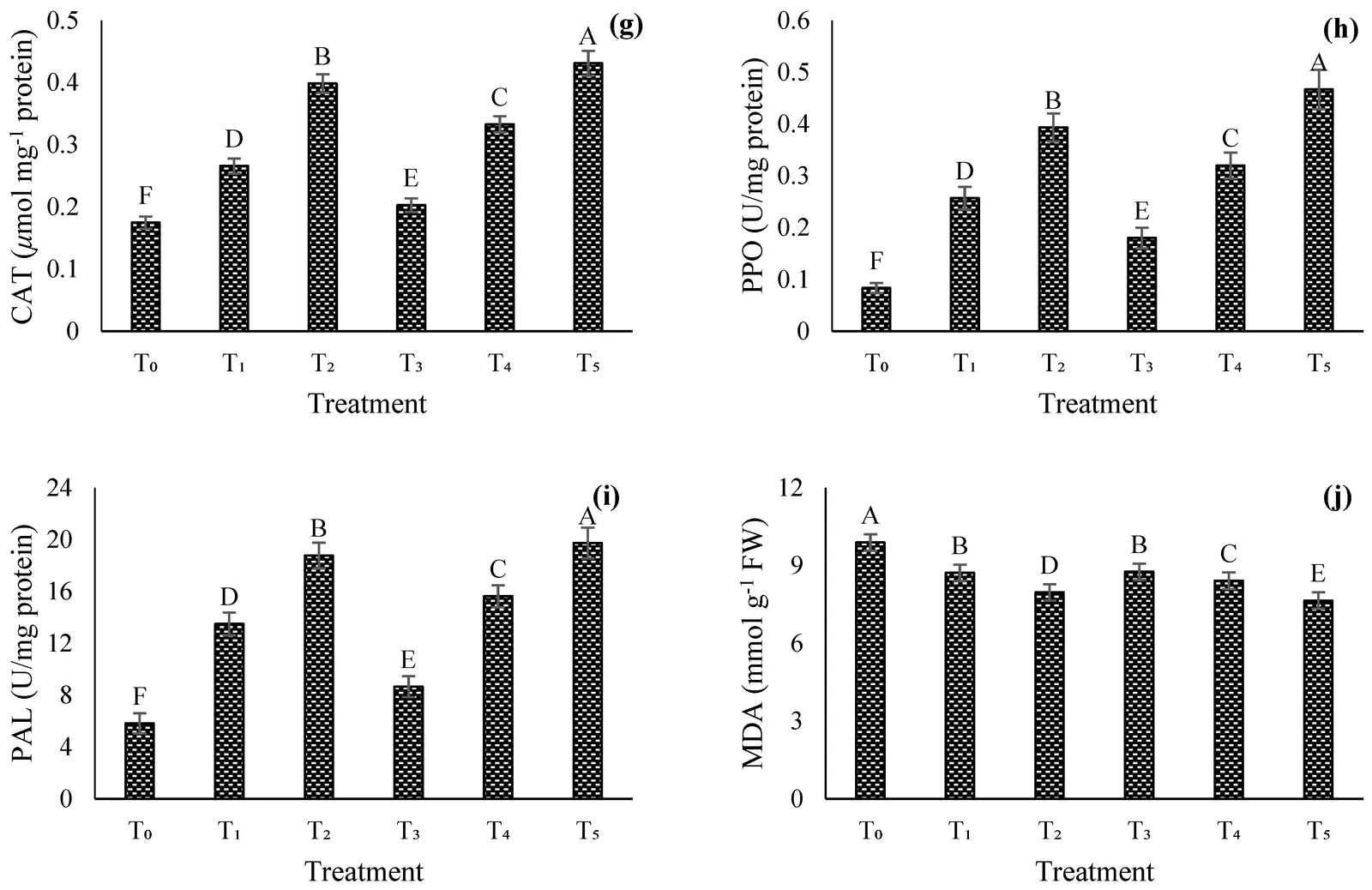
Impact of various treatments on the biochemical attributes, viz., (**a**) proline contents, (**b**) sugar contents, (**c**) flavonoid contents, (**d**) total phenolic contents, (**e**) SOD, (**f**) POD, (**g**) CAT, (**h**) PPO, (**i**) PAL, and (**j**) MDA of wheat under stress regime. T_0_ = Control, T_1_ = 25 mg L^−1^ selenium, T_2_ = 50 mg L^−1^ selenium, T_3_ = Fungal inoculated wheat plants, T_4_ = Fungus + 25 mg L^−1^ selenium, and T_5_ = Fungus + 50 mg L^−1^ selenium.

The sole (T_1_–T_3_) as well as the combined application of fungus + selenium concentrations (T_4_–T_5_) significantly (*p* ≤ 0.05) impacted the biochemical attributes of wheat plants. Higher proline contents (450.21 mg g^−1^ FW) and sugar contents (41.87 ug g^−1^) were observed where T_5_ was applied, whereas the minimum proline contents (342.58 mg g^−1^ FW) and sugar contents (19.69 ug g^−1^) were assessed in the case of the T_3_ treatment. Regarding the flavonoid contents (mg g^−1^ FW) and total phenolic contents (mg g^−1^ FW), it was observed that the combined application of fungus + 50 mg L^−1^ selenium (T_5_) significantly boosted the flavonoid contents (3.55 mg g^−1^ FW) and total phenolic contents (0.096 mg g^−1^ FW) as compared to other tested treatments. Higher SOD (44.94 U/mg protein), POD (9.40 U/mg protein), CAT (0.43 μmol mg^−1^ protein), PPO (0.46 U/mg protein), and PAL contents (19.74 U/mg protein) were observed where T_5_ was applied as compared to other tested treatments over control, whereas reduced MDA contents (7.64 mmol g^−1^ FW) were assessed in the case of the T_5_ treatment; the control exhibited (9.89 mmol g^−1^ FW) contents (Figure 4).

### 3.6. PCA and Correlation Matrix

**Figure 5:**
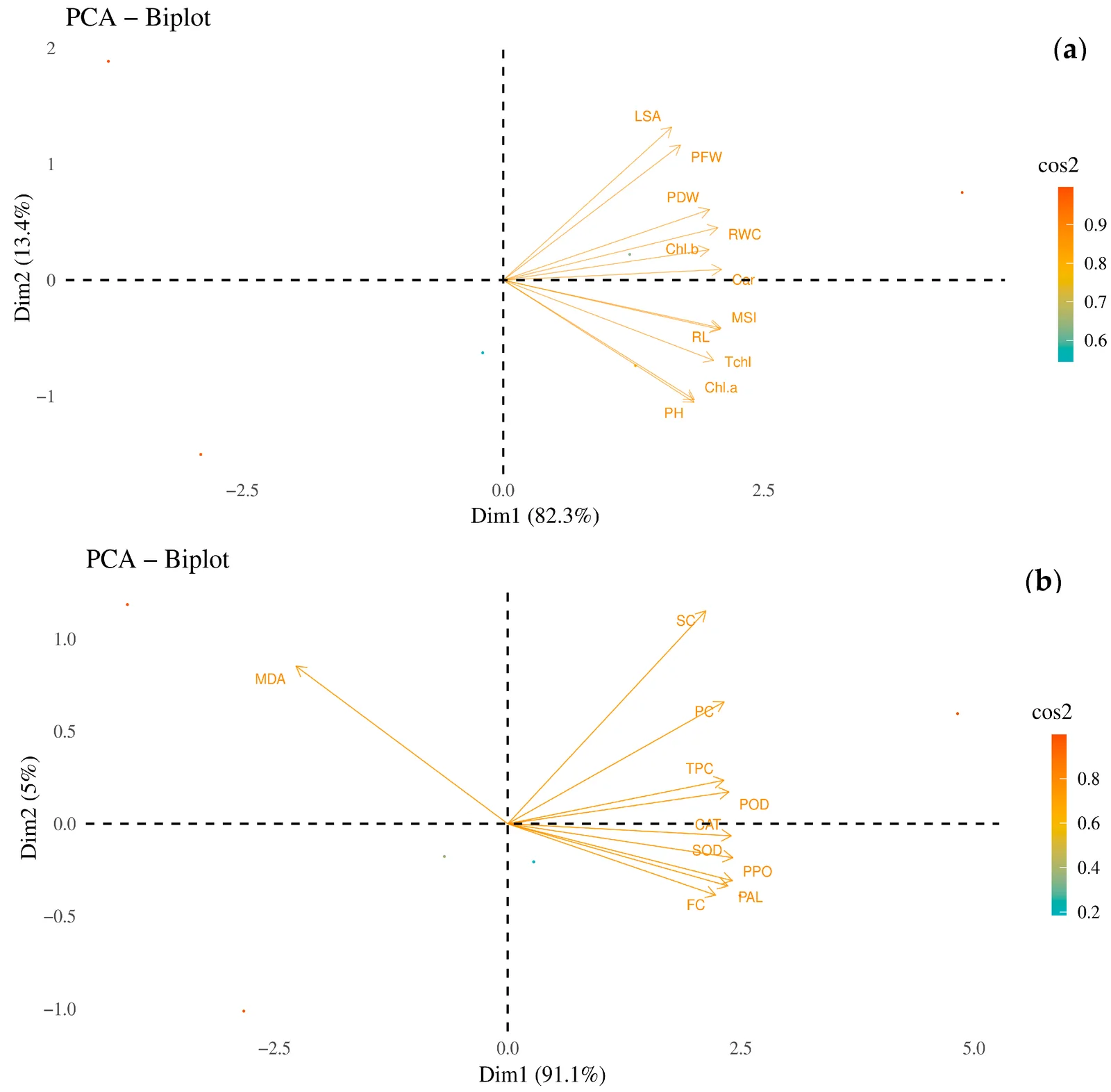
Principal component analysis plot—(**a**) growth and physiological attributes, and (**b**) biochemical attributes—showing correlations between variables for wheat treated with various treatments. Chl a indicates chlorophyll a, PH indicates plant height, Tchl indicates total chlorophyll, RL indicates root length, SC indicates sugar contents, MSI indicates membrane stability index, PC indicates proline contents, Chl b indicates chlorophyll b, Car indicates carotenoid, CAT indicates catalase, POD indicates peroxidase, PAL indicates phenylalanine ammonia lyase, RWC indicates relative water contents, PPO indicates polyphenol oxidase, SOD indicates superoxide dismutase, TPC indicates total phenolic contents, PDW indicates plant dry weight, FC indicates flavonoid contents, PFW indicates plant fresh weight, and LSA indicates leaf surface area.

**Figure 6:**
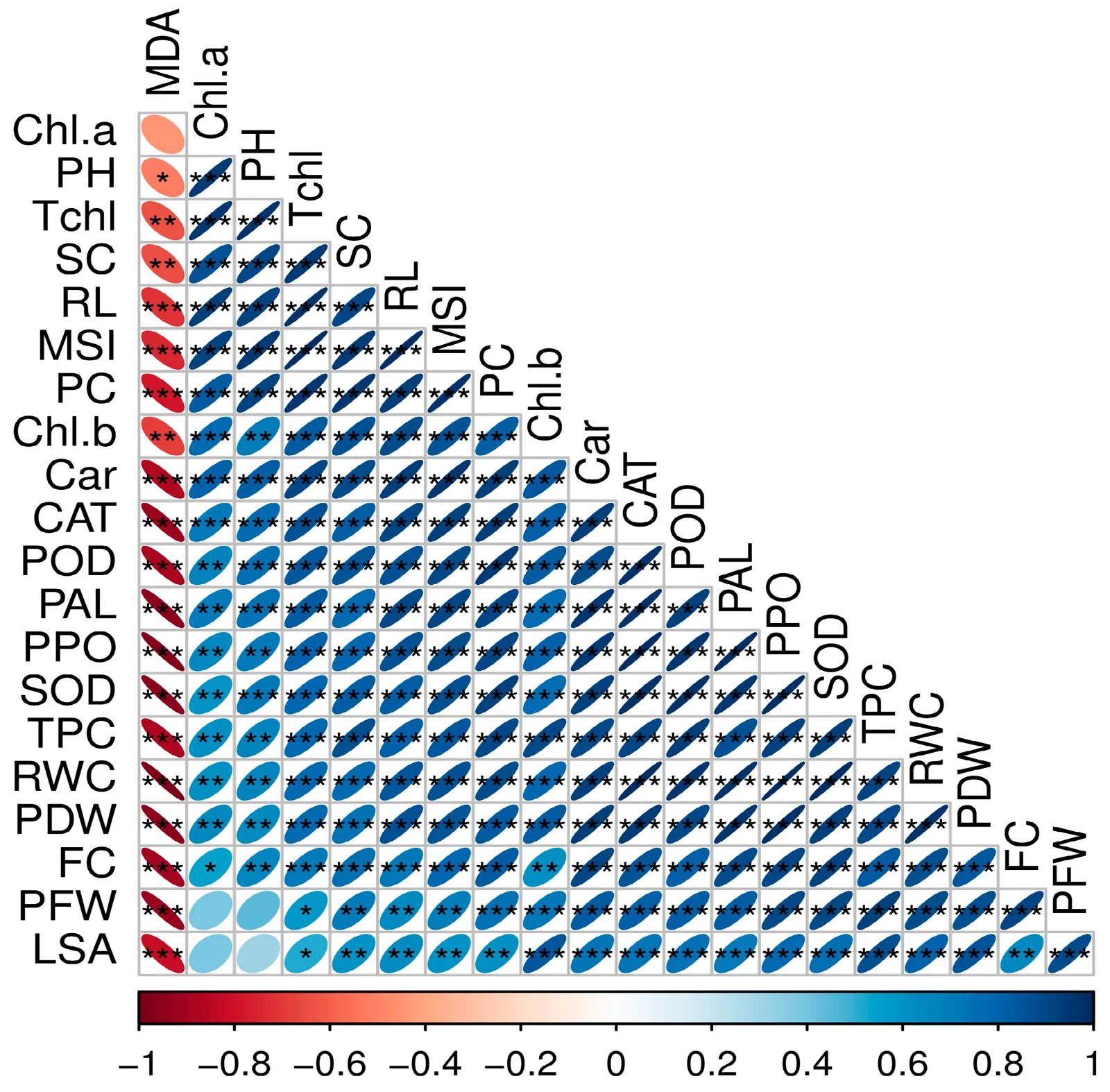
Correlation matrix of various traits of wheat plants treated with various treatments. Chl a indicates chlorophyll a, PH indicates plant height, Tchl indicates total chlorophyll, RL indicates root length, SC indicates sugar contents, MSI indicates membrane stability index, PC indicates proline contents, Chl b indicates chlorophyll b, Car indicates carotenoid, CAT indicates catalase, POD indicates peroxidase, PAL indicates phenylalanine ammonia lyase, RWC indicates relative water contents, PPO indicates polyphenol oxidase, SOD indicates superoxide dismutase, TPC indicates total phenolic contents, PDW indicates plant dry weight, FC indicates flavonoid contents, PFW indicates plant fresh weight, and LSA indicates leaf surface area. *, **, and *** = significant at *p* ≤ 0.05, 0.01, and 0.001, respectively.

Principal component analysis (PCA) was executed to identify the combination pattern between the measured traits, viz., growth, biomass, and physiological and biochemical features in response to the application of various treatments (T_0_–T_5_). The main components in growth and physiological attributes, i.e., Dim1 and Dim2, accounts for 95.7 percent of the total database. Dim1 contributed 82.3% (x-axis) and Dim2 contributed 13.4% (y-axis) of the database (Figure 5a). The main components in biochemical attributes, i.e., Dim1 and Dim2, accounts for 96.1 percent of the total database. Dim1 contributed 91.1% (x-axis) and Dim2 contributed 5% (y-axis) of the database (Figure 5b). To establish the credibility of the observations made in wheat plants, an examination was carried out on the growth, biomass and physio-chemical traits to find out the significant and non-significant correlation. MDA showed a negative relationship with all the attributes, and the remaining attributes were significantly correlated with each other (Figure 6). Chl a exhibited non-significant correlation with MDA. Similarly, PFW and LSA exhibited a non-significant correlation with Chl a and PH.

### 3.7. Heatmap Analysis

**Figure 7:**
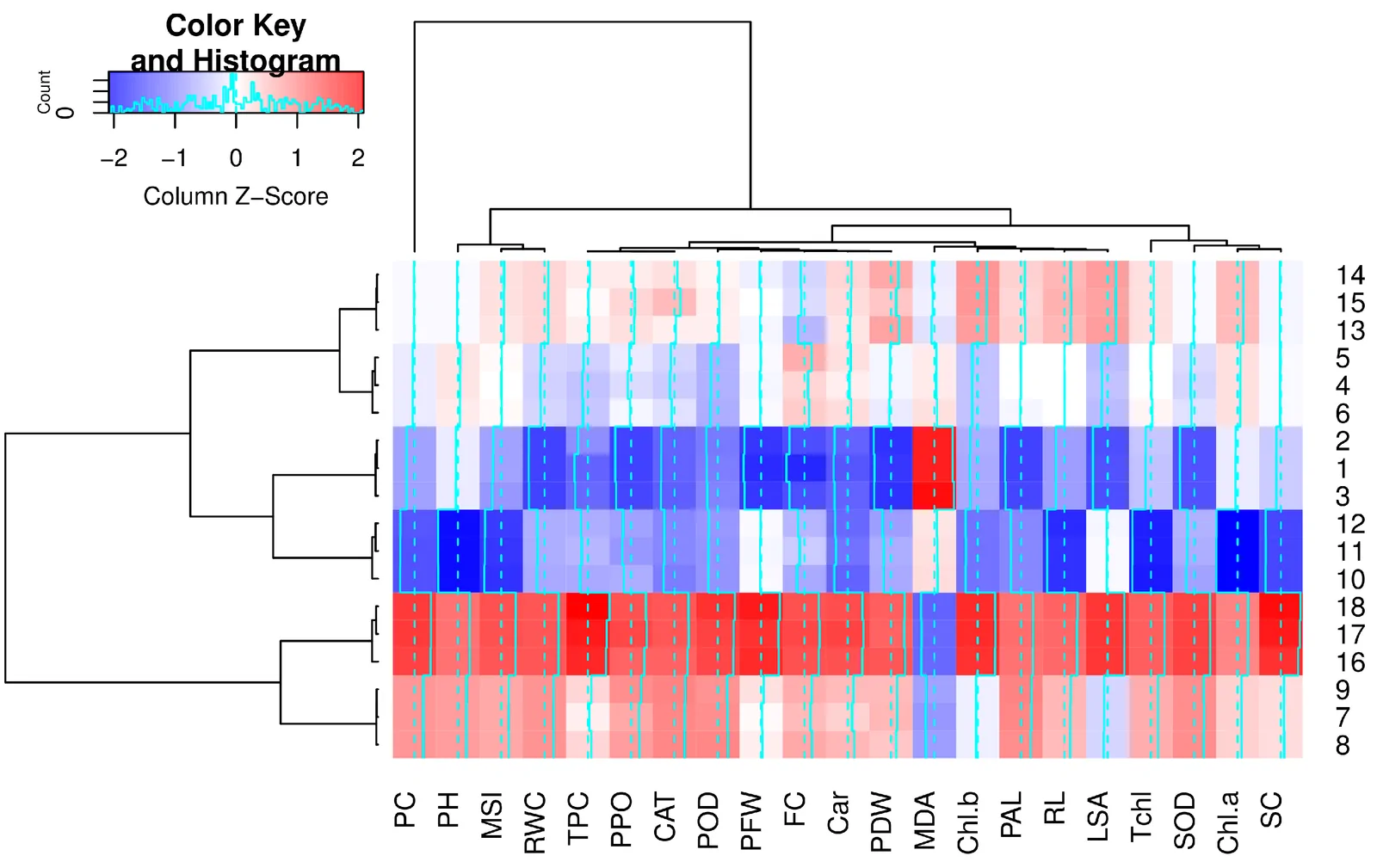
Heatmap presenting response of wheat plants treated with various treatments. PH indicates plant height, PFW indicates plant fresh weight, PDW indicates plant dry weight, LSA indicates leaf surface area, RL indicates root length, Chl a indicates chlorophyll a, Chl b indicates chlorophyll b, Tchl indicates total chlorophyll, Car indicates carotenoid, RWC indicates relative water contents, MSI indicates membrane stability index, PC indicates proline contents, SC indicates sugar contents, FC indicates flavonoid contents, and TPC indicates total phenolic contents.

The cluster heatmap analysis was used to summarize the responses of the plant growth, biomass, and physiological and biochemical characteristics of the wheat plants under the sole and combined application of fungus + selenium concentration (Figure 7). In the context of trait association, the heat map divided the tested treatments (T_0_–T_5_) into various dendrograms. In column, 1 to 12 (T_0_–T_3_) showed the sole treatments, i.e., control, selenium concentrations (25 and 50 mg L^−1^), and fungal-inoculated wheat plants without selenium application, respectively, while 13 to 18 (T_4_–T_5_) showed combinations of fungus with all tested concentrations of selenium. The differential relations of all the characters ranged from positive to negative limits with respect to the used treatments as presented in Figure 7.

## 4. Discussion

Selenium has been suggested to modulate the plant’s secondary metabolism, leading to the production of phytoalexins and other defense-related compounds [30]. These compounds contribute to the plant’s resistance against pathogens, including fungi [13]. Additionally, Se’s effects on plant hormone regulation, particularly the jasmonic acid (JA) and salicylic acid (SA) pathways, could influence the plant’s ability to respond to fungal attacks [52]. 

The current trial describes the resilient antifungal activity of Se against the *B. sorokiniana* (Figure 1). The findings of the current research have indicated that Se can influence plant–fungal interactions by affecting various physiological (Figure 3) and biochemical processes (Figure 4). It was observed that a higher concentration of Se leads to the maximum inhibition of fungal spp., which might be due to the oxidative stress in fungal cells. Similar findings were also observed by [53]. Similar findings were reported by [54], who stated that silver nanoparticles improve the antioxidant defense systems that can tolerate controlled levels of reactive oxygen species (ROS); fungal cells are more sensitive to oxidative stress. At elevated concentrations, it can disrupt the fungal cell’s redox balance, leading to the accumulation of ROS that cause damage to cellular components such as lipids, proteins, and DNA [55]. This oxidative damage can interfere with essential cellular functions and disrupt fungal growth. Changes in membrane integrity affect nutrient uptake and waste expulsion, disrupting vital cellular functions [56]. Furthermore, Se’s impact on fungal cell signaling pathways, such as calcium signaling, may disrupt fungal responses to environmental cues and stressors, further hampering growth and development [57].

It was obvious from the findings of the current study that the application of Se showed a positive impact on plant growth and morphological parameters under both control and fungal inoculation treatments (Figure 2). The improvement in the growth and morphological attributes might be due to its role in several metabolic processes, including antioxidant defense systems, enzyme activation, and hormone regulation [58]. Adequate Se levels can enhance plant health, promote proper root and shoot development, and contribute to increased chlorophyll synthesis, thereby positively influencing photosynthesis and overall growth [59]. It was noticed that fungal infections lead to stunted growth due to resource allocation towards defense mechanisms; Se supplementation might help counteract these effects to some extent. Se-enhanced antioxidant capacity can alleviate cellular damage and allow plants to allocate more resources to growth, leading to improved morphological parameters [60].

It was noticed from the findings that the addition of fungal infections leads to a reduced photosynthetic efficiency due to compromised leaf health and altered chlorophyll synthesis. Se imparts its role as an antioxidant, which becomes significant here, as it can help maintain chlorophyll levels and protect the photosynthetic machinery from oxidative damage [61]. The Se contribution in reducing oxidative stress may enable plants to sustain photosynthetic activity to a greater extent, even in the presence of fungal infection [62]. The findings of the previous studies depicted that Se has been shown to enhance cell membrane stability by reducing lipid peroxidation and maintaining membrane fluidity [63]. This can be particularly relevant in the context of fungal stress, where Se’s membrane-stabilizing effects can contribute to the plant ability to withstand pathogen-induced membrane disruptions [64]. Se showed the protective role in enhancing antioxidant defense, maintaining membrane stability [65], regulating hormone signaling, and potentially influencing nutrient dynamics collectively contribute to the plant’s ability to mitigate the negative impacts of fungal stress [58].

In the present study, wheat subjected to biotic stress showed improved biochemical parameters such as proline, soluble sugar, phenolics, and flavonoids with different levels of Se (25 and 50 mg L^−1^). Fungal infections trigger stress responses that often lead to an increase in proline accumulation [58]. The enhancement in these attributes might be due to the Se effects on plant biochemical parameters under fungal inoculation treatments revolving around its ability to modulate antioxidant metabolism, promote the synthesis of defense compounds, influence signaling molecules like NO, and impact enzyme activities and metabolic pathways [3,66]. The combined action of Se’s antioxidative properties and its potential to redirect metabolic pathways enhances the plant’s ability to manage oxidative stress, sustain its biochemical equilibrium, and mediate effective defense responses against fungal pathogens [67,68]. It is possible that Se might modulate the balance between biochemical attributes and other stress response mechanisms [58]. This could result in altered biochemical attributes in Se-treated plants compared to those without Se supplementation. Se supplementation has been associated with an increase in the synthesis of secondary metabolites, including phytoalexins [69]. Phytoalexins are compounds that plants produce in response to microbial attacks, and they possess antifungal properties [20]. By promoting the production of these defense compounds, Se assists wheat in mounting a stronger defense against fungal pathogens, even under inoculation conditions [58].

In summary, Se’s ability to enhance antioxidant defenses, mitigate oxidative stress, promote the synthesis of defense compounds, facilitate osmotic adjustment, and modulate hormone signaling collectively contributes to its positive effects on wheat biochemical parameters under both control and fungal inoculation treatments. The specific outcomes can vary based on factors like Se concentration, the type of fungal pathogen, and the overall physiological state of the wheat plants. The integration of Se supplementation as part of an integrated disease management strategy holds potential for improving wheat’s resilience against fungal infections. These findings can be used to investigate the processes behind Se supplementation’s benefits and create practical applications for crop protection and enhancement in heterogeneous environments under different stress circumstances. Furthermore, representative photos showing the symptoms of the greenhouse diseases were not available, an acknowledged limitation for this study.

## 5. Conclusions

The present investigation elucidated the capacity of Se to elicit resistance in wheat varieties susceptible to leaf spot disease. In the in vitro study, Se demonstrated a notable antifungal potential against *B. sorokiniana*, surpassing the efficacy of conventional fungicides that were associated with adverse effects. In response to the spot blotch disease-induced stress, wheat plants exhibited promising growth, and physiological and biochemical responses, following the exogenous foliar application of Se (50 mg mL^−1^) that offers greater promise and practical use in a sustainable as well as eco-friendly disease management approach. The induction of resistance by Se was attributed to the stimulation of and non-enzymatic antioxidant defense system of the plant, including the production of proline, phenolic compounds, SOD, POD, CAT, PPO, PAL, and flavonoids. The present study, however, was limited to a single wheat cultivar and narrow range of selenium concentrations in a greenhouse environment. More research with several wheat varieties, field evaluations, yield assessments, and comparisons to standard fungicides are required before practical recommendations can be made for large-scale wheat production.

## Figures and Tables

**Table 1:** Overall experimental layout.

Sr. No.	Treatment	Concentrations (mg L^−1^)
1	T_0_	Control
2	T_1_	Control + Na_2_SeO_3_ (25 mg L^−1^)
3	T_2_	Control + Na_2_SeO_3_ (50 mg L^−1^)
4	T_3_	Fungus Inoculated Wheat
5	T_4_	Pathogen + Na_2_SeO_3_ (25 mg L^−1^)
6	T_5_	Pathogen + Na_2_SeO_3_ (50 mg L^−1^)

**Table 2:** Selected rating scale to find the susceptibility of leaf spot blotch disease.

Sr. No.	Observed Spots on Wheat Leaves (%)	Rating
1	No symptoms observed	No disease
2	1 to 5	Moderately resistant
3	6 to 20	Moderately resistant
4	21 to 40	Moderately susceptible
5	41 to 60	Moderately susceptible
6	>61	Susceptible

## Data Availability

The data supporting the findings of this study are available upon request from the corresponding author. The data are not publicly available due to privacy or ethical restrictions.

## Abbreviations

The following abbreviations are used in this manuscript:

CAT	Catalase
FC	Flavonoid contents
FW	Fresh weight
g	Grams
LSA	Leaf surface area
MDA	Malondialdehyde contents
Mg	Milligrams
MSI	Membrane stability index
PAL	Phenylalanine ammonia-lyase
PC	Proline contents
PCA	Principal component analysis
PDW	Plant dry weight
PFW	Plant fresh weight
POD	Peroxidase
PPO	Polyphenol oxidase
SC	Sugar contents
Se	Selenium
SOD	Superoxide dismutase
TPC	Total phenolic contents
